# Conservative management of breast cancer in the elderly in a developing country

**DOI:** 10.1186/1477-7819-5-108

**Published:** 2007-10-01

**Authors:** Lukas J Wasserman, Justus P Apffelstaedt, Jacobus de V Odendaal

**Affiliations:** 1 Breast clinic, Department of Surgery, University of Stellenbosch, Tygerberg, Cape Town, South Africa

## Abstract

**Background:**

The cost effective treatment of cancer in developing countries remains challenging. In the elderly with possible limited life expectancy, the health expenditure associated with standard treatment regimes should be carefully considered. We present the results of conservative management of breast cancer in the aged in a resource-limited environment.

**Methods:**

Patients aged 70 or older with early breast cancer were treated with tumour excision or simple mastectomy and adjuvant tamoxifen. The records of patients presenting to the Breast Unit between January 1990 and December 2004 were retrieved and demographic, clinical, pathological and oncological data were reviewed. Survival statistics were calculated using the life table method.

**Results:**

A total of 483 patients above 70 years of age were identified. One hundred and eighty eight patients were managed according to the conservative protocol. Forty-one had a simple mastectomy and 147 tumour excision. Their mean age was 77.3 years. The mean follow-up is 62 months. Thirty-one patients (16.4%) were not compliant with tamoxifen use. TNM staging was 0 in 4 patients, I in 42 patients, II in 116 patients and III in 26 patients. There was no 30-day mortality. The cumulative incidence of local recurrence was 3.3% at 5 and 10 years. The cumulative incidence of regional recurrence was 3.3% at 5 years and 4.5% at 10 years. The cumulative incidence of distant recurrence was 6.2% at 5 years and 12.2% at 10 years. The cumulative overall, disease specific and disease free survival at 10 years was 59%, 88% and 81% respectively.

**Conclusion:**

Limited surgery and tamoxifen provide excellent control of breast cancer in the elderly in a resource restricted environment. Radiotherapy and axillary dissection and can be safely omitted thereby reducing health care resource utilization.

## Background

The treatment of breast cancer in the elderly is controversial [1-6]. The life expectancy of elderly patients in developing countries may be less than in developed countries [7] and the health expenditure associated with standard treatment regimes should be carefully considered.

Some authors from developed countries recommend less aggressive options in the elderly [2,8,9], while others advocate standard treatment for all patients regardless of age [3,5,9].

With increasing age, the incidence of well-differentiated cancers and special histopathological subtypes associated with a favourable prognosis increases [8]. The potential for local recurrence decreases with age [10-12].

Due to these factors and an increased death rate from concomitant disease, it may be possible to avoid the morbidity associated with axillary dissection and radiotherapy in the elderly without increasing the risk of local and regional recurrence and survival [10,12-16].

We present here an update of a prior audit of conservative management of breast cancer in the aged in a resource-limited environment [17] with emphasis on the relationship of pathological data to patterns of local and distant failure. To our knowledge this is the only data on breast cancer outcomes in similar circumstances.

## Methods

In patients aged 70 or older, tumour excision or simple mastectomy for small breasts and adjuvant tamoxifen for oestrogen receptor positive tumours was performed. Patients with oestrogen receptor positive T3 and T4b lesions are considered for neo-adjuvant tamoxifen in an attempt to shrink the tumour before surgery. Neither radiotherapy nor axillary dissection is offered, even in patients with N1 nodal status.

The records of 483 women aged 70 or older at the time of diagnosis that had presented to the Breast Unit between January 1990 and December 2004 were reviewed. The records of patients that were treated with limited surgery and tamoxifen were analyzed for the variables summarized in Table 1. Data were collected from a prospective breast cancer database.

**Table 1:** Data collected

**Clinical**
Co-morbidity
TNM staging
Treatment
Early mortality
Compliance with tamoxifen
**Histopathology**
Pathological size in millimetres
Histological grading [51]
Evidence and extent of ductal carcinoma in situ
Evidence of lymphovascular infiltration
Resection margins (clear = >3 mm, marginal = 1–3 mm, involved = <1 mm)
**Survival data**
Time to local, regional, or distant recurrence
Current status
Disease-free survival
Disease specific survival
Overall survival

Clinical staging was according to the sixth edition of the Union International Contre le Cancer/American Joint Committee on Cancer TNM classification manual[18]. Resection of all gross tumour was performed in all patients. Tumours were analyzed for the pathological variables summarized in Table 1. Due to resource restrictions, hormone receptor and c-erb B2 status were not determined at our institution before March 2004. Consequently, these variables are only known on a few elderly patients with early breast cancer.

Telephone follow-up was conducted for all patients not seen at the hospital within the preceding 6 months. Deceased patients were classified as either dead from disease or dead of other causes based on information from the primary care physician, caregivers or family members. Survival statistics were calculated using the life table method [19].

## Results

Out of a total of 3681 patients diagnosed with breast cancer at Tygerberg Hospital, 483 patients were above 70 years of age. Two hundred and thirty three tumours (48%) were considered too advanced for the protocol. Of these, eight had stage IIA disease, 20 had Stage IIB disease, and 22 had stage IIIA disease, 75 had stage IIIB disease and 108 patients presented with stage IV disease. Of the 125 patients with stage II and III disease, 85 were excluded due to bulky axillary disease requiring a full axillary dissection and 40 due to the advanced nature of their breast lesions requiring radiation therapy for local control. Thirty five patients (7.2%) were considered too frail for any surgery. In nine (1.9%) patients other treatment options were chosen: One patient had palpable axillary nodes and received a full axillary clearance as due to extremely poor socio-economic circumstances compliance with a regular follow-up regime was impossible. Four patients were partially treated elsewhere before referral to our centre. One patient had postoperative radiation for a multicentric tumour. One patient requested a modified radical mastectomy. Two patients had squamous cell carcinomas and were excluded from the study. Fourteen patients had either postoperative radiotherapy (2 patients) or a modified radical mastectomy as part of international multicenter trials. Four patients refused surgery. One hundred and eighty eight patients were treated according to the protocol. Forty-one had a simple mastectomy (SM) and 147 tumour excisions (TE).

Age ranged from 70 to 95 years. The mean age was 77.3 years. Associated co-morbidities were common with a mean of 1.4 co morbidities per patient. Table 2 and 3 reflect these co morbidities.

**Table 2:** Number of patients with co morbidities

**Co morbidities**	**Number of Patients**
Hypertension	117
Heart disease	54
Diabetes Mellitus	28
Pulmonary disease	7
Renal disease	2
Stroke	8
Other malignancy	22
Dementia	5
Arthritis	2
Hypothyroidism	3
Schizophrenia	1
Peripheral vascular disease	2

**Table 3:** Number of co morbidities per patient

**Number of Co morbidities**	**Number of Patients**
5	1
4	4
3	16
2	59
1	73

The clinical TNM classification of tumours is reflected in Table 4 and 5.

**Table 4:** T-stage of tumours

**T- stage**	**Tumour excision (n = 147)**	**Mastectomy (n = 41)**
Tis	4 (3%)	0
T1b	10 (7%)	0
T1c	30 (22%)	3 (8%)
T2	85 (58%)	13 (33%)
T3	10 (7%)	12 (30%)
T4b	8 (5%)	13(33%)

**Table 5:** TNM- stage of tumours

**TNM- stage**	**Number of Patients**	**%**
0	4	2%
I	42	22%
II	116	62%
III	26	14%

Twenty-one patients (14%) treated by TE and ten patients (24%) treated by SM had small, mobile nodes palpable.

The World Health Organization tumour types are reflected in Table 6[20]. Grossly tumour free margins were achieved in all patients. In 126 patients, histopathological margins were more than 3 mm. In 38 patients, margins were less than 3 mm and in 24 patients resection margins were involved with tumour on microscopic examination; no further resections were performed to achieve tumour free margins. The mean tumour size for patients who had a TE was 22.2 mm (range: 5–70 mm) and 47.25 mm for those who had a simple mastectomy (range: 10–150 mm).

**Table 6:** World Health Organization tumour type [20]

**Type**	**Number of Patients**	**%**
Ductal carcinoma in situ	4	2
Infiltrating ductal		
Total	136	72
Grade 1	45	24
Grade 2	74	39
Grade 3	17	9
Infiltrating lobular	14	7
Mucinous	26	14
Papillary	6	3
Medullary	2	1

One hundred tumours (53%) had no in situ component. Thirty four tumours had extensive ductal carcinoma in situ, 24 in the TE group and ten in the SM group. Lymphovascular infiltration was detected in 16 (9%) of tumours. There was no 30-day mortality.

The mean follow-up for the entire group is 62 months (range 4 to 166 months). Five are alive with evidence of disease at 43, 62, 75, 95 and 128 months respectively. Twenty patients have died of breast cancer, 93 are alive with no evidence of disease, and 53 have died of other diseases. Of the 188 patients, 17 are lost to follow-up. Thirty-one patients (16.4%) were not compliant with tamoxifen use.

None of the patients who had a SM and 6 patients with a TE suffered a local recurrence. In four of these patients disease was controlled with a further local excision at 7, 23, 27 and 50 months after initial treatment. Two of these patients have died of intercurrent illnesses. They were disease free at the time of death. The other two patients are alive and well with no evidence of disease at 55 and 62 months after initial treatment. One patient's local recurrence was controlled with radiotherapy 8 months after initial treatment. The patient is alive with no evidence of disease at 43 months. One patient had concurrent regional metastases and was treated by mastectomy with axillary dissection at 55 months. This patient went on to develop distant metastases at 86 months and died of breast cancer at 166 months.

All 6 patients had infiltrating ductal carcinoma. In 3 patients resection margins were clear. In 2 they were marginal and in 1 the margins were involved. The patient with involved margins developed a recurrence at 8 months after initial surgery and is discussed above. None of the patients with local recurrence had tumours with an extensive in situ component.

The patient that also developed regional and distant metastasis had a grade 2 infiltrating ductal carcinoma with lymphovascular infiltration. None of the other patients with local recurrence had tumours with lymphovascular infiltration. The mean tumour size of the 6 tumours was 14.8 mm (range 10 mm–20 mm)

None of the patients that were non compliant with tamoxifen use developed a local recurrence.

**Figure 1:**
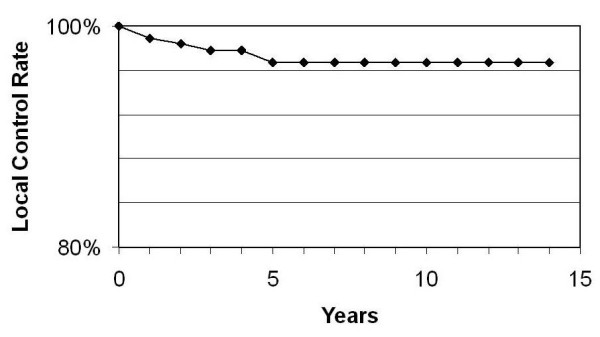
Local control rate.

The cumulative incidence of local recurrence is 3.3% at 5 and 10 years. The local control rate is reflected in Figure 1.

**Figure 2:**
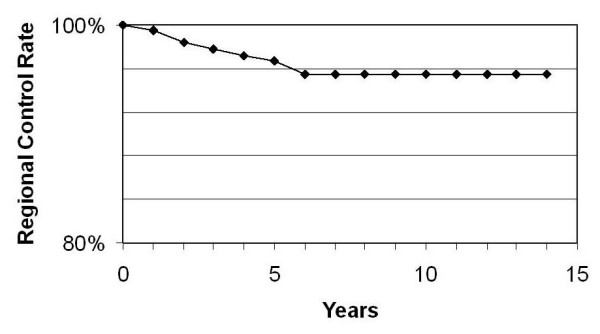
Regional control rate.

Regional recurrence developed in one patient who had a SM at 47 months. Her recurrence was controlled by axillary dissection. She developed distant metastases and died 67 months after initial diagnosis of breast cancer. Eight patients who had a tumour excision developed regional recurrence at 4, 19, 23, 35, 45, 55, 64 and 112 months respectively. In 6 patients the recurrence was controlled by axillary dissection. Two of these patients went on to develop distant metastases. The one had a regional recurrence at 35 months and distant metastases at 96 months and died shortly afterwards. The other patient was already discussed. One patient refused axillary dissection when she developed a regional recurrence at 23 months. She died of intercurrent illness at 75 months. One patient was considered too frail for any intervention when she developed regional recurrence at 112 months and died of intercurrent illness 7 months later. The other 4 are alive with no evidence of disease. The cumulative incidence of regional recurrence is 3.3% at 5 years and 4.5% at 10 years. The regional control rate is reflected in Figure 2. None of the patients with palpable nodes at the time of diagnosis required axillary dissection for regional control during the course of their follow up. Two of the patients that had a TE and suffered a local recurrence were not compliant with tamoxifen use.

**Figure 3:**
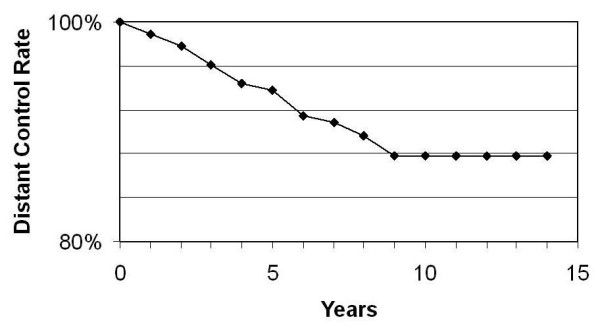
Distant control rate.

Distant metastases developed in 16 patients who had a TE (10.8%) and in 5 patients who had a SM (12.2%) i.e. in 21 of the whole group. One patient developed bone metastases at 83 months and is still alive with disease at 95 months after initial diagnosis; 20 died of metastatic breast cancer. The cumulative incidence of distant recurrence is 6.2% at 5 years and 12.2% at 10 years. The distant control rate is reflected in Figure 3.

Of the patients that developed distant recurrence, 15 had infiltrating ductal carcinoma, three had papillary carcinoma, two had mucinous carcinoma, and one had infiltrating lobular carcinoma. Of the patients with infiltrating ductal carcinoma, three had grade 1 tumours, ten had grade 2 tumours and two had grade 3 tumours. One patient that developed a distant recurrence was not compliant with tamoxifen use.

**Figure 4:**
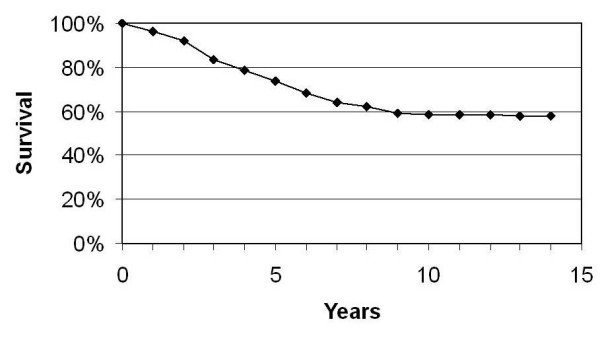
Overall survival.

**Figure 5:**
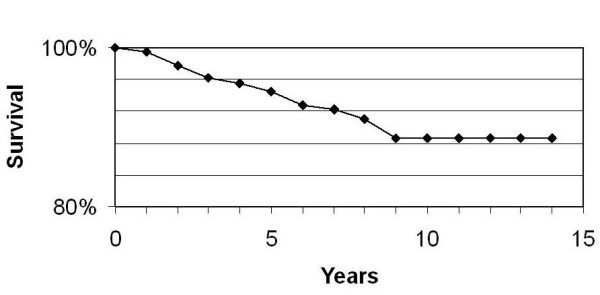
Disease specific survival.

**Figure 6:**
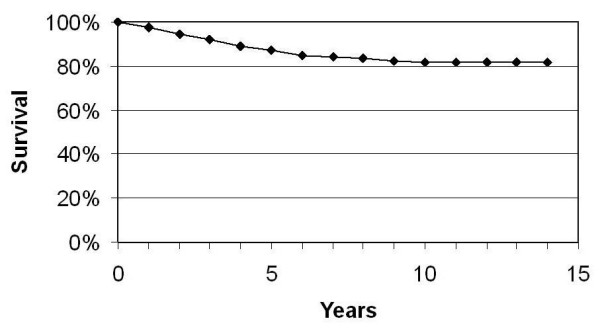
Disease free survival.

The cumulative overall survival, disease specific survival and disease free survival rates are reflected in Figures 4 to 6.

## Discussion

In developed countries more than a third of all breast cancers occur in women over the age of 70 years [1,2,4]. In a report from South Africa it is apparent that the disease is seen at an earlier age in Blacks (mean 49 years) and Coloureds (mean 53 years) than in Whites (mean 60 years). In this series 15% of Coloured patients, 5% of Black patients and 30% of White patients were older than 70 years at presentation [21]. The population that we serve is predominantly Coloured.

Breast cancer in the elderly is a more indolent disease than in younger women [1,4,16]. In an analysis of the histopathological findings in 1869 post menopausal women with breast cancer in a developed country, Fisher *et al*., demonstrated more favourable features with increasing age. These features include less vascular invasion and lymphoplasmacytic stromal reaction. Elderly woman also have a higher frequency of hormone receptor positive tumours (85% oestrogen receptor positive versus 67% in younger patients). Fifty eight percent (compared with 50%) were diagnosed with node negative disease although 20% had tumours >5 cm (versus 13% of those younger than 65). The incidence of grade 3 tumours also decreased with age [8]. These results are comparable to the low rates of lymphovascular invasion (9%) and grade 3 tumours (9%) in our series. In a series of 572 mastectomy specimens of women of all ages from Pakistan, similar pathological features were found as in western series of non-screened populations [22].

The potential for local recurrence decreases with age [10-12], an effect enhanced by tamoxifen [23]. The addition of irradiation to partial mastectomy in younger patients reduces the incidence of ipsilateral recurrence without affecting survival [24-27].

In a large Italian series evaluating breast conservation in women of all ages, radiotherapy did not improve local control rates in patients over the age of 65 years [12]. The results of three trials evaluating the role of radiotherapy in early breast cancer in the elderly are summarized in Table 7. These results are comparable to our data.

**Table 7:** Recurrence and survival rates with omission of radiotherapy.

**Authors**	**Inclusion age**	**Number of patients**	**Local or regional recurrence**		**Local recurrence**		**Regional recurrence**		**Overall survival**	
Hughes et al [15]	>70	636	L+T	4%					L+T	86%
			L+T+R	1% (P < 0.001)					L+T+R	87% (P = 0.94)
Fyles et al [52]	> 50	769			L+T	7.7%	L+T	2.5%	L+T	93.2%
					L+T+R	0.6% (P < 0.001)	L+T+R	0.5% (p = 0.049	L+T+R	92.8% (p = 0.83)
Gruenberger et al [10]	>60	356			Q+ A ± C ± T	5.4%			Q+ A ± C ± T	82%
					Q+ A ± C ± T + R	5.8%			Q+ A ± C ± T +R	82.7%

L +T : Lumpectomy and Tamoxifen. L+T+R : Lumpectomy, Tamoxifen and whole breast irradiation

Q: Quadrenectomy. A: axillary clearance R: whole breast irradiation C; CMF. T: Tamoxifen

Even in developed countries elderly women often do not receive radiotherapy: In women older than 60 years treated for stage I or II breast cancer with segmental mastectomy, only 39.2% were referred for postoperative radiotherapy, whereas 82% of similar patients less than 60 years were referred. In elderly patients that received radiation therapy, the complications were minimal. There was a significant benefit of irradiation in achieving local and regional control. The data even suggested that postoperative irradiation may be more beneficial when extended to elderly patients post segmental mastectomy than among younger women. These results are in contrast to most other series evaluating radiotherapy in the elderly and these differences are difficult to explain. No analysis was however done of factors other than age (i.e. co-morbidities, receptor status and tamoxifen use) as reasons for referral for radiotherapy. A considerable number of patients not referred for radiotherapy also had tumours with unknown resection margin status [9].

In our patients the cumulative incidence of local recurrence is 3.3% at 5 and 10 years. It compares favourable to reported local recurrence rates in younger patients receiving radiation after tumour excision [27-32]. This demonstrates the value of appropriate patient selection. The resection of infiltrated axillary nodes does not have a controlling influence on survival but provides prognostic information for planning post surgical therapy. A further role for axillary dissection is that of regional control [13,14,27,33,34].

Martelli *et al*., [13] reported on 321 elderly patients with operable breast cancer and clinically non-palpable axillary nodes randomised to surgery with (S+AD) or without axillary dissection (S). All patients received tamoxifen. There were no significant differences in overall or breast cancer mortality or cumulative incidence of breast events between the two groups. The authors concluded that elderly patients with small tumours without clinical axillary involvement may be satisfactorily treated with conservative surgery and tamoxifen.

Twenty three percent of patients who had an axillary dissection had metastatic nodal infiltration. However, only 1.8% of patients who had tumour excision only required a delayed axillary dissection for clinically overt axillary disease. These findings suggest that the vast majority of microscopic axillary nodal metastases are clinically irrelevant.

In our patients the prognostic information supplied by axillary dissection would not have altered management. Due to resource limitations, before March 2004 patients received tamoxifen irrespective of hormone receptor status and no chemotherapy was offered. In our series 16.4% of patients had palpable axillary nodes. It stands to reason that the nodes were microscopically involved in many more cases. However, the cumulative incidence of regional recurrence is comparable to that observed by Martelli *et al*., [13] mentioned above. The fact that none of the patients with palpable nodes required axillary dissection for regional control also confirms the effectiveness of tamoxifen in controlling these nodes.

Adjuvant tamoxifen treatment substantially improves the survival of women with oestrogen receptor positive tumours [35]. Tamoxifen significantly reduced the rate of treatment failure at local and distant sites, second breast cancers and the incidence of tumour recurrence after lumpectomy and breast irradiation [36,37]. Tamoxifen has also been evaluated as sole treatment of breast cancer in the elderly and earlier studies suggested that tamoxifen alone is sufficient treatment [38-40]. Recent meta – analysis however shows that breast cancer specific survival and progression-free survival is worse in women treated with tamoxifen alone [41-45]. We support the notion that tamoxifen alone is inadequate therapy that should be reserved for very frail patients.

Compliance with Tamoxifen use was not evaluated in any of the above mentioned series. One of the reasons for the low rate of treatment failure in general of our patients may be the use of adjuvant tamoxifen and the high rate of long term compliance with its use.

The use of tamoxifen in patients with unknown or negative hormone receptors is debatable as it may expose patients to the side effects of the drug without any clinical benefit [35]. The probability of tumours in the elderly being oestrogen receptor positive has been reported as 85% [8]. The cost of hormone receptor determination at our institution is about ten times that of one month's supply of tamoxifen. It can therefore be argued that it is not cost effective to determine receptor status in view of isolating the 15% of patients who will not benefit from tamoxifen. Despite its adverse effects tamoxifen is well tolerated in the elderly and might have an advantageous effect on blood lipid levels and bone mineral density [4,8,42-44,46,47].

By 2010, the majority of approximately 1.5 million annual new cases of breast cancer will be diagnosed in women in countries with limited resources [48]. The challenges of treating these patients are multiple.

Adjuvant systemic and radiation therapies are increasingly expensive and careful consideration of cost-efficacy is needed.

The further life expectancy of South African women between 70 and 74 is 10.4 years, of women 75 to 79 it is 8 years and for those 80 to 84 it is 5.9 years versus in the USA 14.8 years, 11.2 years and 8.5 years respectively [7]. This shows that even in a developing country elderly women may have a substantial further life expectancy and treatment options should be considered carefully as to not compromise local or distant disease control.

Compared with the treatment of early breast cancer, the treatment of advanced breast cancer is more resource intensive and has poorer outcomes. This highlights the potential benefit of earlier detection and diagnosis, especially in countries with limited resources [49].

In a survey investigating the awareness of breast and cervical cancers among South African women, almost one fifth of the women had not heard of these cancers, and almost half were unaware of the breast self-examination technique. Lower awareness levels were also found in older and rural women [50]. In our study 48.2% of patients presented with disease too advanced for conservative treatment, demonstrating the need for earlier detection of breast cancer in developing countries.

## Conclusion

In a country with limited resources, limited surgery and tamoxifen provide excellent control of breast cancer in the elderly comparable to results obtained in less resource restricted environments.

## Competing interests

The author(s) declare that they have no competing interests.

## Authors' contributions

LJW collected the data, performed the statistical analysis and drafted the manuscript. JPA conceived of the study, and participated in its design and coordination. JDO collected some of the data and performed some of the statistical analysis. All the authors read and approved the final manuscript.

## References

[B1] Aapro M, Piccart M (1998). Breast cancer. Crit Rev Oncol Hematol.

[B2] Aapro MS (2002). Progress in the treatment of breast cancer in the elderly. Ann Oncol.

[B3] Extermann M (2004). Management issues for elderly patients with breast cancer. Curr Treat Options Oncol.

[B4] Law TM, Hesketh PJ, Porter KA, Lawn-Tsao L, McAnaw R, Lopez MJ (1996). Breast cancer in elderly women: presentation, survival, and treatment options. Surg Clin North Am.

[B5] Singletary SE, Shallenberger R, Guinee VF (1993). Breast cancer in the elderly
1. Ann Surg.

[B6] Wanebo HJ, Cole B, Chung M, Vezeridis M, Schepps B, Fulton J, Bland K (1997). Is surgical management compromised in elderly patients with breast cancer?. Ann Surg.

[B7] World Health Organization (2004). World Health Organization statisctics: Life Tables. http://www.who.int/whosis.

[B8] Fisher CJ, Egan MK, Smith P, Wicks K, Millis RR, Fentiman IS (1997). Histopathology of breast cancer in relation to age. Br J Cancer.

[B9] Kantorowitz DA, Poulter CA, Sischy B, Paterson E, Sobel SH, Rubin P, Dvoretsky PA, Mishalak W, Doane KL (1988). Treatment of breast cancer among elderly women with segmental mastectomy or segmental mastectomy plus postoperative radiotherapy. Int J Radiat Oncol Biol Phys.

[B10] Gruenberger T, Gorlitzer M, Soliman T, Rudas M, Mittlboeck M, Gnant M, Reiner A, Teleky B, Seitz W, Jakesz R (1998). It is possible to omit postoperative irradiation in a highly selected group of elderly breast cancer patients. Breast Cancer Res Treat.

[B11] Nemoto T, Patel JK, Rosner D, Dao TL, Schuh M, Penetrante R (1991). Factors affecting recurrence in lumpectomy without irradiation for breast cancer
1. Cancer.

[B12] Veronesi U, Marubini E, Mariani L, Galimberti V, Luini A, Veronesi P, Salvadori B, Zucali R (2001). Radiotherapy after breast-conserving surgery in small breast carcinoma: long-term results of a randomized trial
4. Ann Oncol.

[B13] Martelli G, DePalo G, Rossi N, Coradini D, Boracchi P, Galante E, Vetrella G (1995). Long-term follow-up of elderly patients with operable breast cancer treated with surgery without axillary dissection plus adjuvant tamoxifen. Br J Cancer.

[B14] Greco M, Agresti R, Raselli R, Giovanazzi R, Veronesi U (1996). Axillary dissection can be avoided in selected breast cancer patients: analysis of 401 cases. Anticancer Res.

[B15] Hughes KS, Schnaper LA, Berry D, Cirrincione C, McCormick B, Shank B, Wheeler J, Champion LA, Smith TJ, Smith BL, Shapiro C, Muss HB, Winer E, Hudis C, Wood W, Sugarbaker D, Henderson IC, Norton L (2004). Lumpectomy plus tamoxifen with or without irradiation in women 70 years of age or older with early breast cancer. N Engl J Med.

[B16] Benhaim DI, Lopchinsky R, Tartter PI (2000). Lumpectomy with tamoxifen as primary treatment for elderly women with early-stage breast cancer. Am J Surg.

[B17] Odendaal JV, Apffelseadt JP (2003). Limited surgery and tamoxifen in the treatment of elderly breast cancer patients.. World J Surg.

[B18] Singletary SE, Greene FL (2003). Revision of breast cancer staging: the 6th edition of the TNM Classification. Semin Surg Oncol.

[B19] Bland M (1995). Life-table Analysis and Kaplan-Meier Graph.. An Introduction to Medical Statistics.

[B20] Organization WH, World Health Organization (1981). Histological typing of breast tumours No 2.

[B21] Hacking EA, Dent DM, Gudgeon CA (1984). Malignant tumours of the breast. Frequency distribution by age, race and stage at Groote Schuur Hospital, Cape Town, 1971-1981
1. S Afr Med J.

[B22] Siddiqui MS, Kayani N, Sulaiman S, Hussainy AS, Shah SH, Muzaffar S (2000). Breast carcinoma in Pakistani females: a morphological study of 572 breast specimens
4. J Pak Med Assoc.

[B23] Fisher B, Dignam J, Bryant J, Wolmark N (2001). Five versus more than five years of tamoxifen for lymph node-negative breast cancer: updated findings from the National Surgical Adjuvant Breast and Bowel Project B-14 randomized trial
5. J Natl Cancer Inst.

[B24] (2000). Favourable and unfavourable effects on long-term survival of radiotherapy for early breast cancer: an overview of the randomised trials. Early Breast Cancer Trialists' Collaborative Group. Lancet.

[B25] Fisher B, Jeong JH, Bryant J, Anderson S, Dignam J, Fisher ER, Wolmark N (2004). Treatment of lymph-node-negative, oestrogen-receptor-positive breast cancer: long-term findings from National Surgical Adjuvant Breast and Bowel Project randomised clinical trials. Lancet.

[B26] Fisher B, Anderson S (1994). Conservative surgery for the management of invasive and noninvasive carcinoma of the breast: NSABP trials. National Surgical Adjuvant Breast and Bowel Project
2. World J Surg.

[B27] Lichter AS, Lippman ME, Danforth DN, d'Angelo T, Steinberg SM, deMoss E, MacDonald HD, Reichert CM, Merino M, Swain SM, . (1992). Mastectomy versus breast-conserving therapy in the treatment of stage I and II carcinoma of the breast: a randomized trial at the National Cancer Institute
3. J Clin Oncol.

[B28] Fisher B, Redmond C, Poisson R, Margolese R, Wolmark N, Wickerham L, Fisher E, Deutsch M, Caplan R, Pilch Y, . (1989). Eight-year results of a randomized clinical trial comparing total mastectomy and lumpectomy with or without irradiation in the treatment of breast cancer
2. N Engl J Med.

[B29] Blichert-Toft M, Rose C, Andersen JA, Overgaard M, Axelsson CK, Andersen KW, Mouridsen HT (1992). Danish randomized trial comparing breast conservation therapy with mastectomy: six years of life-table analysis. Danish Breast Cancer Cooperative Group
1. J Natl Cancer Inst Monogr.

[B30] Arriagada R, Le MG, Rochard F, Contesso G (1996). Conservative treatment versus mastectomy in early breast cancer: patterns of failure with 15 years of follow-up data. Institut Gustave-Roussy Breast Cancer Group
1. J Clin Oncol.

[B31] van Dongen JA, Bartelink H, Fentiman IS, Lerut T, Mignolet F, Olthuis G, van der SE, Sylvester R, Winter J, van Zijl K (1992). Randomized clinical trial to assess the value of breast-conserving therapy in stage I and II breast cancer, EORTC 10801 trial
1. J Natl Cancer Inst Monogr.

[B32] Jacobson JA, Danforth DN, Cowan KH, d'Angelo T, Steinberg SM, Pierce L, Lippman ME, Lichter AS, Glatstein E, Okunieff P (1995). Ten-year results of a comparison of conservation with mastectomy in the treatment of stage I and II breast cancer
1. N Engl J Med.

[B33] Fisher B, Redmond C, Fisher ER, Bauer M, Wolmark N, Wickerham DL, Deutsch M, Montague E, Margolese R, Foster R (1985). Ten-year results of a randomized clinical trial comparing radical mastectomy and total mastectomy with or without radiation. N Engl J Med.

[B34] Veronesi U, Orecchia R, Zurrida S, Galimberti V, Luini A, Veronesi P, Gatti G, D'Aiuto G, Cataliotti L, Paolucci R, Piccolo P, Massaioli N, Sismondi P, Rulli A, Lo SF, Recalcati A, Terribile D, Acerbi A, Rotmensz N, Maisonneuve P (2005). Avoiding axillary dissection in breast cancer surgery: a randomized trial to assess the role of axillary radiotherapy. Ann Oncol.

[B35] (1998). Tamoxifen for early breast cancer: an overview of the randomised trials. Early Breast Cancer Trialists' Collaborative Group. Lancet.

[B36] Cummings FJ, Gray R, Tormey DC, Davis TE, Volk H, Harris J, Falkson G, Bennett JM (1993). Adjuvant tamoxifen versus placebo in elderly women with node-positive breast cancer: long-term follow-up and causes of death. J Clin Oncol.

[B37] Fisher B, Costantino J, Redmond C, Poisson R, Bowman D, Couture J, Dimitrov NV, Wolmark N, Wickerham DL, Fisher ER, . (1989). A randomized clinical trial evaluating tamoxifen in the treatment of patients with node-negative breast cancer who have estrogen-receptor-positive tumors
2. N Engl J Med.

[B38] Bates T, Riley DL, Houghton J, Fallowfield L, Baum M (1991). Breast cancer in elderly women: a Cancer Research Campaign trial comparing treatment with tamoxifen and optimal surgery with tamoxifen alone. The Elderly Breast Cancer Working Party. Br J Surg.

[B39] Gazet JC, Ford HT, Coombes RC, Bland JM, Sutcliffe R, Quilliam J, Lowndes S (1994). Prospective randomized trial of tamoxifen vs surgery in elderly patients with breast cancer. Eur J Surg Oncol.

[B40] Robertson JF, Ellis IO, Elston CW, Blamey RW (1992). Mastectomy or tamoxifen as initial therapy for operable breast cancer in elderly patients: 5-year follow-up
8. Eur J Cancer.

[B41] Mustacchi G, Ceccherini R, Pluchinotta A, De Matteis A, Maiorino L, Farris A, Scanni A, De Placido S (2002). Results of adjuvant treatment in breast cancer women aged more than 70: Italian cooperative group experience
1. Tumori.

[B42] Love RR, Mazess RB, Barden HS, Epstein S, Newcomb PA, Jordan VC, Carbone PP, DeMets DL (1992). Effects of tamoxifen on bone mineral density in postmenopausal women with breast cancer. N Engl J Med.

[B43] Love RR (1994). Tamoxifen in healthy premenopausal and postmenopausal women: different risks and benefits. J Natl Cancer Inst.

[B44] Powles TJ, Hickish T, Kanis JA, Tidy A, Ashley S (1996). Effect of tamoxifen on bone mineral density measured by dual-energy x-ray absorptiometry in healthy premenopausal and postmenopausal women. J Clin Oncol.

[B45] Hind D, Wyld L, Beverley CB, Reed MW (2006). Surgery versus primary endocrine therapy for operable primary breast cancer in elderly women (70 years plus). Cochrane Database Syst Rev.

[B46] Akhtar SS, Allan SG, Rodger A, Chetty UD, Smyth JF, Leonard RC (1991). A 10-year experience of tamoxifen as primary treatment of breast cancer in 100 elderly and frail patients. Eur J Surg Oncol.

[B47] Cummings FJ, Gray R, Davis TE, Tormey DC, Harris JE, Falkson G, Arseneau J (1985). Adjuvant tamoxifen treatment of elderly women with stage II breast cancer. A double-blind comparison with placebo. Ann Intern Med.

[B48] Love RR, Love SM, Laudico AV (2004). Breast cancer from a public health perspective
2. Breast J.

[B49] Eniu A, Carlson RW, Aziz Z, Bines J, Hortobagyi GN, Bese NS, Love RR, Vikram B, Kurkure A, Anderson BO (2006). Breast cancer in limited-resource countries: treatment and allocation of resources
1. Breast J.

[B50] Pillay AL (2002). Rural and urban South African women's awareness of cancers of the breast and cervix
2. Ethn Health.

[B51] Bloom HJC, Richardson WW (1957). Histological grading in breast cancer.. Br J Cancer.

[B52] Fyles AW, McCready DR, Manchul LA, Trudeau ME, Merante P, Pintilie M, Weir LM, Olivotto IA (2004). Tamoxifen with or without breast irradiation in women 50 years of age or older with early breast cancer. N Engl J Med.

